# Men’s knowledge and involvement on obstetric danger signs, birth preparedness and complication readiness in Burayu town, Oromia region, Ethiopia

**DOI:** 10.1186/s12884-019-2661-4

**Published:** 2019-12-21

**Authors:** Addisu Gize, Alemtsehay Eyassu, Balkachew Nigatu, Mekonen Eshete, Nebiyou Wendwessen

**Affiliations:** 1 Department of Microbiology, St. Paul’s Hospital Millennium Medical College, Addis Ababa, Ethiopia; 2 Department of Psychiatry, St. Paul’s Hospital Millennium Medical College, Addis Ababa, Ethiopia; 3 Department of Gynecology and Obstetrics, St. Paul’s Hospital Millennium Medical College, Addis Ababa, Ethiopia; 4 Department of Surgery, School of Medicine, College of Health Sciences, Addis Ababa University, Addis Ababa, Ethiopia; 5 Institute for Health Care Improvement, Addis Ababa, Ethiopia

**Keywords:** Male involvement, Birth preparedness and complication readiness, Burayu and Ethiopia

## Abstract

**Background:**

Men’s involvement in obstetrics care is an important strategy in reducing preventable maternal morbidity and mortality. This is particularly important in developing countries where men often make decision on financial, health and other family issues. Hence, the objective of this study was to assess men’s knowledge and involvement in obstetric danger signs; birth preparedness and complication readiness in Burayu town administration, Oromia, Ethiopia.

**Methods:**

A community based cross-sectional study was conducted in Burayu town administration, Oromia Region from May 2016 to July 2016. Multistage with systematic random sampling techniques were employed. Bivariate and multivariate logistic regression analyses were performed using SPSS version 20. *P*-value less than 0.05 were taken as a cutoff point to declare significant association.

**Result:**

A total of 523 men were involved in the study. The mean and ± SD age of the study participant was 36.6 ± 7 years. Majority of the participants were Orthodox religion followers and, employees of private organization, regarding residency majority were residing in urban setting.

Pregnancy related vaginal bleeding was the most familiar danger sign recognized by the study participants which was 342(65.4%).

From the total, 441(84.3%) of men were highly involved in preparation of arranging for postpartum cultural food expenses, 345(66.0%) for clean clothes both for the baby and mother; and 71–76% participants were involved in availing transport money for antenatal, delivery and postnatal care. The study revealed that educational status of men, monthly income, knowledge of pregnancy danger sign, delivery and post-delivery care, and knowledge of birth preparedness and complication readiness (BP/CR) were significantly associated with men’s involvement in BP/CR.

**Conclusion:**

Majority of participants had knowledge on obstetric danger sign. Men showed low interest to donate blood to their wives during antenatal, delivery and postpartum care. There is a need continued awareness creation on danger sings as well as birth preparedness.

## Introduction

The global maternal mortality ratio decreased from 385 deaths per 100, 000 live births in 1990, to 216 in 2015; However, it is still huge challenge in sub-Saharan Africa countries [1].

The average Maternal Mortality Ratio (MMR) of industrialized countries is 8 per 100,000 live births. When we compare this figure with Ethiopia which has an estimated MMR of 412 per 100,000 live births [2], it shows a marked difference and makes the country one of the greatest contributors of maternal deaths in absolute numbers worldwide [1–3].

It is reported that maternal deaths account for 21% of all deaths, a figure that is amongst the highest in the world [3, 4]. The major causes of maternal deaths are obstructed/prolonged labour (13%), ruptured uterus (12%), severe preeclampsia/ eclampsia (11%) and malaria (9%). Moreover, 6% of all maternal deaths were attributable to complications from abortion [5, 6].

Several studies have reported that lack of advance planning to have a skilled birth attendant during delivery, and poor or low understanding of birth preparedness (BP) and complication readiness (CR) contribute to these negative maternal outcomes. To overcome this, the family and community at large need to work on advanced planning and preparation to ensure safety and wellbeing of mothers and newborns throughout pregnancy, delivery and post-delivery.

According to several studies in Ethiopia, the most common causes of maternal mortality in Ethiopia in the last decade were obstructed labor/uterine rupture, hemorrhage, hypertensive disorders of pregnancy, and sepsis/infection [3, 7]. Maternal mortality can be partly reduced by increasing awareness on the danger signs of obstetric complications and involving husbands in birth preparedness practice [8]. All listed causes are preventable by intervention at antenatal care, intrapartum and post-partum care. Among the antepartum cares, birth preparedness and complication readiness plans are the most important.

Birth preparedness includes educating the mother and her family to recognize the normal signs of labor. Most pregnant women and their families do not know how to recognize the danger signs of complications; though, every pregnancy is considered at risk. Birth preparedness is one of the main strategies in implementing safe pregnancy programs. The components of birth preparedness are knowledge on the danger signs of obstetric complications and emergencies; choosing a preferred birthplace and attendant at birth; making arrangements with the attendant at birth and arranging transport for birth. According to a study in Mekele, northern Ethiopia, the awareness of the concept of BP/CR was high, but recognition of key danger signs was poor. This study recommended that birth preparedness and complication readiness should focus not only on frequency of antenatal care but also in the contents of health education given during ANC follow-ups [9].

In developing countries, men are important actors who have significant influence both directly and indirectly on reproductive health outcomes of women and children [10]. A study done in southern Ethiopian found that husbands who had awareness of danger signs of obstetric complications were two times more likely to be involved in birth preparedness practice than respondents who had no awareness of danger signs of obstetric complications [8]. Very few studies have been conducted in Ethiopia to evaluate the involvement of husbands in BP/CR during pregnancy and child birth. This also rings true for the site of this study, Burayu town, which is located just outside of the capital city of Addis Ababa and represents both an urban and rural community.

Thus, the aim of this study was to assess men’s’ knowledge and involvement on obstetrical danger signs, birth preparedness, complication readiness as well as factors which influence these outcomes among husbands of women who gave birth in the last 12 months prior to the study.

## Materials and methods

### Study settings

A cross-sectional study was conducted in the community of Burayu town administration from May 2016 to July 2016. Husbands of women who gave birth in the last 12 months prior to the study were enrolled. Burayu is located in Oromia National Regional State, 15 km from Addis Ababa. The population of Burayu town administration is estimated to be 156,463, of which 51% are women. The estimated total number of women of reproductive age and women who gave birth within the last 12 months in the town are 17,926 and 3161 respectively (Data from town administration health office). The town administration has two health centers, serving an estimated 32,596 estimated households. All the health centers and health posts give maternal health services.

### Source population

All husbands/ partners of women in reproductive age who have at least one child and living in Burayu town administration, Oromia region, Ethiopia.

### Study population

Husbands/ partners of women in reproductive age who delivered 12 months prior to the study period in the selected kebeles of Burayu town administration, Oromia region, Ethiopia.

### Inclusion and exclusion criteria

Husbands/ partners who reside in the study area 1 year prior to the study and whose wives gave birth with in12 months preceding the survey participated in the study. Whereas those who were unable to give consent, were too ill to undergo an interview, and lived apart from their wives were excluded from the study.

### Sample size determination

The formula for calculating the sample size was:

$$ n=\frac{{Z^2}_{1-\raisebox{1ex}{$\alpha $}\!\left/ \!\raisebox{-1ex}{$2$}\right.}p\left(1-p\right)}{d^2} $$

Where; *n* is the desired sample size, *p* is proportion of husbands/ partners of women who are involved in birth preparedness from the previous study in Mekele town, Tigray region, Ethiopia [10], *Z*^*2*^_*1-α/*2_ was critical value at 95% CI (1.96), *d* is the margin of error between the sample and the proportion. Calculation of the sample size for both husband’s knowledge of obstetric (OB) danger signs and involvement were compared, and the result of involvement in BP/CR was taken because of the maximum figure, which was account 524 sample size, considering a design effect of 1.5 and a non-response rate of 10%.

### Sampling procedure

Multistage sampling was carried out in Burayu town. The town administration is constituted by six kebeles (the smallest administrative structure in Ethiopia which has its own health post according to the Ethiopian health system). To get representative and adequate sample kebeles, five kebeles were selected using simple random sampling from the total six kebeles. By using each health post in a kebele as a reference point, the total sample size was allocated to each selected kebeles through proportionally and systematically random sampling method applied to select the study subject.

We used all directions from this reference point (North, South, East and West directions as far as suitable roads were available for systematic random sampling method application).

From the houses which were included under the sampling interval, one house was selected using random sampling method. A systematic random sampling was applied to the next household using the calculated every K^th^ interval in every kebele. For absent study subjects, rescheduling was done to conduct the study. However, if the selected household does not fulfill the inclusion criteria, the next household was substituted for our study, and if more than one candidate was available in the single house hold, one of them was interviewed through a lottery method.

### Data collection tools and procedure

A structured questionnaire was used which contained sections; socio-demographic, obstetric, BP/CR and knowledge on key obstetric danger signs (Additional file 1).

The English version of the questionnaire was translated to Amharic and Afan Oromo was then back translated to English. The questionnaire was pre-tested before the actual study on a similar characteristic residing outside of the study area. The pre-test findings were discussed with data collectors and some amendments were done on the questionnaire. Data collection was administered by five female Health Extension Workers (HEWs) who were fluent in both Amharic and Afan Oromo. One supervisor with BSc nursing background supervised the data collectors. The data collectors underwent 2 days of training to become familiar with questionnaire undergo practical exercise on the study instrument and data collection procedures. Data were collected through face-to-face interview with the study subjects.

Men’s involvement on birth preparedness and complication readiness was dependent variable. Whereas, socio-demographic variables, Health Extension Workers (HEWs) influence, obstetric/service utilization of a wife; perceived quality of care provided; accessibility of health facility; husband/ partner individual factor; awareness on danger sign of obstetric complication were considered as independent factors.

### Operational definitions

#### Men’s involvement on birth preparedness and complication readiness

A husband / partner of a woman was considered well prepared or involved if he was found to have made arrangements for at least three of the component practices of BP/CR (identified place of delivery, identified skilled provider, saved money, identified transport ahead of emergency and identified blood donor) for his pregnant wife.

#### Knowledgeable on danger signs of pregnancy

A husband was considered well knowledgeable- if he mentioned at least two danger sign of pregnancy (vaginal bleeding, swollen hand/ face, blurred vision).

#### Knowledgeable on danger signs of labor/childbirth

A husband was considered well knowledgeable on danger signs of labor/childbirth- if he could mention at least three of the key four danger signs for labor/childbirth (vaginal bleeding, prolonged labor (> 12 h), convulsion, retained placenta).

### Data management

Data were checked for completeness every day and entered into Epi-Info version 7.0 statistical software and cleaned thoroughly before exported to SPSS version 20 for further analysis. Corrections were made and the cleaned data were exported from Epi Info version 7.0 to SPSS version 20 for analysis.

### Data analysis procedures

Univariate and bivariate logistic regression analysis were used. Binary logistic analysis used to determine the association between the independent and outcome. Multiple logistic regression analysis was used to control confounding factors. *P*-value less than 0.05 were taken as a statistically significant association.

### Ethical consideration

Prior to the beginning of the study, ethical clearance was obtained from Institutional Review Board of the St. Paul’s Hospital Millennium Medical College. An official letter was obtained from St. Paul’s Hospital Millennium Medical College to the respective city administration/study setting. Permission from the city administration health bureau was secured to carry out the study. Informed written consent was obtained from each individual respondent. The information collected from study participant was kept confidential.

## Results

A total of 523 men were involved in the study. Most of the participants were employees of private organization, residing in urban areas, and 199 (37.9%) were orthodox religion followers. The mean and ± SD age of the study participant was 36.6 ± 7 years. The mean and ± SD monthly income of the house hold was 3452 ± 2093 Ethiopian Birr; majority of house hold had one child (Table 1).

**Table 1:** Background characteristics of men’s knowledge and involvement in recognizing danger signs, birth preparedness and complication readiness in Burayu town, Oromia, Ethiopia, 2016, *N* = 523

Socio-demographic characteristics	Category	Number (%)
Age in years	≤ 29	101(19.3)
	30–39	295 (56.4)
	40+	127(24.3)
Religion	Orthodox	198(37.9)
	Protestant	192(36.7)
	Muslim	106(20.3)
	Other	27(5.2)
Ethnicity	Oromo	390(74.6)
	Amhara	69(13.2)
	Others	64(12.2)
Occupational Status	Private employee	179(34.2)
	Civil servant	147(28.1)
	Farmer	138(26.4)
	Merchant	59(11.3)
Educational status	Illiterate	69(13.2)
	Primary school (1-8th)	108(20.7)
	Secondary school (9-12th)	135(25.8)
	Diploma and above	211(40.3)
Monthly house hold income	≤1000	170(32.5)
	1001–3000	206(39.4)
	3001–5000	109(20.8)
	≥5000	38(7.3)
Number of wives	One	502(96.0)
	Two	20(3.8)
	Three	1(0.2)
Number of currently living children	None	5(1.0)
	One	159(30.4)
	Two	120(22.9)
	Three	98(18.7)
	Four	87(16.6)
	Five and above	54(10.3)
Residence	Rural	114(21.8)
	Urban	409(78.2)
Communication with partner	Good	494(94.5)
	Poor	29(5.5)

During pregnancy, vaginal bleeding was the most recognized danger sign. Arranging for postpartum food expenses was the most commonly mentioned activity for birth preparedness and complication readiness (Table 2).

**Table 2:** Men’s knowledge of obstetrical danger sign, birth preparedness and complication readiness in Burayu, Oromia, Ethiopia, 2016

Knowledge of Danger Signs During Pregnancy		
Danger sign indicators	Yes (%)	No (%)
Convulsion	126(24.1)	397(75.9)
Nausea and Vomiting	150(28.7)	373(71.3)
Severe abdominal pain	92(17.6)	431(82.4)
Vaginal bleeding	342(65.4)	181(34.6)
Swollen leg and face	194(37.1)	329(62.9)
Difficulty of breathing	86(16.4)	437(83.6)
Severe headache	273(52.2)	250(47.8)
Accelerated and reduced fetal movement	99(18.9)	424(81.1)
High grade fever	149(28.5)	364(69.6)
Blurred vision	159(30.4)	365(69.8)
Water breaks without labour	65(12.4)	458(87.6)
Loss of consciousness	142(27.2)	381(72.8)
Knowledge of BP/CR		
Items of BP/CR	Yes (%)	No (%)
Arranging for postpartum cultural food expenses	447(85.5)	76(14.5)
Savings for emergencies	159 (30.4)	364(69.6)
Arrangement for skilled birth assistance	153(29.3)	370(70.7)
Identifying a mode of transportation	333(63.7)	190(36.3)
Identifying decision maker for emergency	128(24.5)	395(75.5)
Identifying place of delivery	340(65.0)	183(35.0)
Arranging blood donors	82(15.7)	441(84.3)
Clean clothes & other materials for Baby/Mother’s	345(66.0)	178(34.0)
Prevention of HIV mother to child	124(23.7)	399(76.3)

Men highly involved in preparing for expenses related to postpartum cultural food expenses, transport or medications needed during antenatal, delivery and postnatal care as well as preparing clean clothes and other materials for the baby or mother. Conversely, the number of men who made pre-arrange for blood donation during delivery was low (Table 3).

**Table 3:** Men’s involvement during pregnancy, delivery and post-delivery in Burayu, Oromi, Ethiopia, 2016

	Yes (%)	No (%)
Preparation for Delivery		
Arranging for postpartum cultural food expenses	441(84.3)	82(15.7)
Savings money for emergencies	166 (31.7)	357(68.3)
Arrangement for skilled birth assistance	161(30.8)	362(69.2)
Identifying a mode of transportation	337(64.4)	186(35.6)
Identifying decision maker for emergency	134(25.6)	389(74.4)
Identifying place of delivery	332(63.5)	191(36.5)
Arranging blood donors	78(14.9)	445(85.1)
Clean clothes & other materials for child /Mother	354(67.7)	169(32.3)
Prevention of HIV mother to child	114(21.8)	409(78.2)
Involvements of Husbands in Antenatal care		
Gives permission only	84(16.1)	439(83.9)
Money for transport/drugs	395(75.5)	128(24.5)
Personally accompanies	321(61.4)	202(38.6)
Donates blood	26(5.0)	497(95.0)
Involvements of Husbands in Delivery		
Gives permission only	102(19.5)	421(80.5)
Money for transport/drugs	388(74.2)	135(25.8)
Personally accompanies	378(72.3)	145(27.7)
Donates blood	40(7.6)	483(92.4)
Involvements of Husbands in Postpartum care		
Gives permission only	82(15.7)	441(84.3)
Money for transport/drugs	376(71.9)	147(28.1)
Personally accompanies	334(63.9)	189(36.1)
Donates blood	23(4.4)	500(95.6)

Factors associated with men’s involvement on birth preparedness and complication readiness included monthly household income (AOR = 0.29, 95% CI: 0.090–0.923), perceived believe of getting appreciation from health workers when the husband accompanies his wife during maternal service (AOR = 7.47, 95% CI: 1.24–44.896), worry for possibility of maternal death during childbirth (AOR = 2.15, 95% CI: 1.072–4.314); knowledge on danger sign during pregnancy (AOR = 0.30,95% CI:0.14–0.599); birth preparedness and complication readiness knowledge related to delivery or post-delivery (AOR = 0.06, 95% CI: 0.03–0.105), (Table 4).

**Table 4:** Factors associated with men’s involvement on birth preparedness and complication readiness in Burayu, Oromia, Ethiopia, 2016

Variables	Category	N (%)	COR at 95%CI	AOR at 95% CI
Respondent’s educational status	1. Illiterate	69(13.2%)	0.33(0.171–0.642)*	0.46 (0.161–1.302)
	2. Primary school (1-8th)	108(20.7%)	0.74(0.458–1.204)	2.05 (0.954–4.394)
	3.Secondary school (9-12th)	135(25.8%)	0.62(0.393–0.983)*	0.86 (0.415–1.786)
	4. Diploma and above	211(40.3%)	1	
Monthly house hold income in Ethiopian Birr	≤1000	170(32.5%)	0.16(0.076–0.358)**	0.74 (0.221–2.457)
	1001–3000	206(39.4%)	0.13(0.060–0.282)**	0.29 (0.090–0.923)*
	3001–5000	109(20.8%)	0.37(0.168–0.823)*	0.37 (0.111–1.219)
	≥5001	38(7.3%)	1	
Believe of who should make decisions about major household purchases	Respondent	39(7.5%)	0.84(0.404–1.728)	0.59 (0.175–1.961)
	Pregnant women herself	62(11.9%)	2.27(1.324–3.885)*	1.73 (0.689–4.342)
	Respondent & wife jointly	422(80.7%)	1	
Believe that pregnancy is only a woman affair	Yes	93(17.8%)	1.19(0.308–4.577)	1.70 (0.252–11.426)
	No	414(79.2%)	2.59(0.728–9.244)	1.12 (0.185–6.801)
	I don’t Know	16(3.1%)	1	
Thinking about the attitude of health workers towards men who accompany their wives to health facility.	Cooperative & welcoming	438(83.7%)	6.44(1.952–21.262)*	1.68 (0.325–8.705)
	Uncooperative & harsh	46(8.8%)	11.00(2.96–40.858)**	7.47 (1.24–44.896)*
	Do not know	39(7.5%)	1	
People talking about men who escort their wives to the health unit	Good and encouraging	415(79.3%)	3.17(1.727–5.814)**	2.10 (0.819–5.383)
	Bad and discouraging	24(4.6%)	0.71(0.187–2.725)	0.32 (0.049–2.060)
	Do not know	84(16.1%)	1	
Belief of any pregnant woman is susceptible to complications during child birth	Yes	406(77.6%)	1.63(0.624–4.254)	1.29 (0.280–5.952)
	No	95(18.2%)	0.62(0.214–1.816)	0.52 (0.098–2.739)
	I don’t Know	22(4.2%)	1	
Possibility of maternal death during child birth	Yes	130(24.9%)	7.05(4.559–10.914)**	2.15 (1.072–4.314)*
	No	393(75.1%)	1	
Knowledge of danger sign of pregnancy, delivery and after delivery	Poor knowledge	299(57.2%)	0.07(0.043–0.106)**	0.30 (0.14–0.599)**
	Good Knowledge	224(42.8%)	1	
Knowledge of BP/CR	Poor Knowledge	347(66.3%)	0.03(0.018–0.049)**	0.06 (0.03–0.105)**
	Good Knowledge	176(33.7%)	1	

*Variables significant at *p* < 0.05, **Variables significant at *p* < 0.001

## Discussion

Though the common direct obstetric causes of maternal mortality are known, the contribution of men’s involvement is unknown in developing country like Ethiopia.

In developing countries including Ethiopia the most crucial factor contributing towards high maternal deaths is the desire to have children. In this study, 93(17.8%) of the participants thought that, it is one of the main issues and 54 (10.3%) desire to have five and more children. The study showed that majority of the men were under 30 years old 101(19.3%) similar to other studies [11, 12]. Nevertheless, well-targeted, focused male involvement programs can have a positive influence on reproductive health behaviors by:- increased birth preparedness, and complication readiness, and communication between partners.

Among the danger signs during pregnancy, the most commonly identified were vaginal bleeding 342 (65.4%), followed by severe headache 273 (52.2%). Respondents’ knowledge on water breaks without labor; difficulty of breathing and severe abdominal pain during pregnancy was low. However, the current participants knowledge of vaginal bleeding and severe headache seem better than from the study conducted in other parts of the country like Southern (42%) and Northern Ethiopia (60%) [10, 13].

Of the components of birth preparedness and complication readiness, 441 (84.3%) of the participants arranged for post-partum cultural food expenses, and 337 (64.4%) of them identified place of delivery. The finding in this study slightly higher than the study done in Mekele, Northern Ethiopia [10]. The least birth preparedness and complication readiness were arranging blood donors 78 (15%) and prevention of HIV transmission from mother to child 124 (23.7%). This finding contradicts a study done in Sothern Ethiopia where preparedness for blood donation and HIV transmission prevention were identified [14]. This might be due to more committed rural health extension workers giving health education for better involvement of men birth preparedness and complication readiness and identification of danger sign [10].

Many of the participants were involved in availing money for transport or medications, 395(75.5%) and the majority of men 321 (61.4%) accompanied their wives to antenatal, delivery and post-delivery services.

Most participants, 332(63.5%) identified place of delivery and 134(25.6%) involved in decision making during emergency. Compared to the study done in Ambo, Western Ethiopia, which showed 80.5% identified a place of delivery and 64.4% involvement were involved in decision making, the findings in the current study are lower [15]. The current study also showed a lower percentage of men arranged for skilled birth assistance 161(30.8%) and 166 (31.7%) saved money for obstetrical emergencies when compared to the study in Ambo [16]. One explanation for this difference might be due to our study area which is found closer to the capital city of the country, Addis Ababa, and the study participants’ proximity to services which are more are more easily accessible, reducing the need to plan for obstetrical care.

Male partners who were illiterate and those who attend primary school only were 0.46 (AOR; 95% CI = 0.161–1.302) and 2.05 (AOR; 95%CI = 0.954–4.394) times less likely respectively to be involved in birth preparedness and complication readiness characteristics compared to respondents with educational status of diploma and above.

Among the socio-demographic factors, monthly house- hold income was significantly associated with male involvement on birth preparedness and complication readiness, i.e., those who had a monthly salary 1001–3000 ETB were 0.29 times (AOR; 95% CI = 0.090–0.923) less likely to be involved in BP/CR when compared to those who earned ≥5000 ETB.

This study also revealed that the attitude of health workers towards men who accompany their wives to health facility, knowledge of danger sign of pregnancy and knowledge of BP/CR specific to delivery and post-delivery period were significant factors associated with men’s involvement in BP/CR. If the participant perceived that the health workers were uncooperative and harsh; they were 7.47times (AOR; 95%CI =1.24–44.896) more likely not to have knowledge of danger sign and be involved in complication readiness. Those who had poor knowledge of danger sign of pregnancy, delivery and post-delivery were 0.30 (AOR; 95% CI = 0.14–0.599), and 0.06 (AOR; 95% CI = 0.03–0.105) times, respectively less involved in the danger sign, birth preparedness and complication readiness as compared to good knowledge.

### Strengths and limitations of the study

The fact that the current study was a community based study gives a major strength for quality of data gathered. Some of the other strengths of this study were; using large number of men who were willing to participate in the study, systematic random sampling technique to select the study subjects; recall period of 1 year to avoid recall bias, and exploring this untouched subject in developing areas where the target groups are very important for decision making. However, using cross-sectional study design, may not be utilized for cause effect analysis. Another limitation may be related to social or informational bias given that the data collectors were health extension workers who live in the same community as the participants and are expected to work on improving knowledge and involvement of men in BP/CR within this community.

## Conclusion

In this study, the majority of the participant’s decisions were made jointly with their wives. Most of the deliveries were uncomplicated and live delivery. Vaginal bleeding was the only obstetrics danger signs recognized by most participants and overall, participants showed low involvement in donating blood for their wives during antenatal, delivery and postpartum care. Participant had knowledge on many of birth preparedness and complication readiness items during pregnancy with the exception of knowledge on decision making for obstetrical emergencies, arranging blood donation and prevention of HIV mother to child.

Our study revealed that educational status of men; monthly household income; attitude of health workers towards men who accompany their wives to health facility were factors significantly associated with knowledge of danger sign of pregnancy, delivery and post-delivery and knowledge of BP/CR.

### Recommendation

We recommend that husbands/partners and the community in general should be educated on obstetrics danger sign and birth preparedness. It is also important to increase men’s awareness on the possible need for blood donation during delivery and postnatal care.

## Supplementary information

**Additional file 1:** Questionnaire for Men’s Knowledge on Obstetric Danger Signs, Birth Preparedness and Complication Readiness.

## Data Availability

The data that support the findings of this study will be available from the corresponding author upon reasonable request in the form of statistical package for social sciences (SPSS).

## Abbreviations

ANC: Ante natal care
BP: Birth Preparedness
CI: Confidence interval
CR: Complication Readiness
FMOH: Federal Ministry of Health
MMR: Maternal Mortality Rate
SPSS: Statistical package for social science
WHO: World Health Organization
