# Supplementary material for: Men’s knowledge and involvement on obstetric danger signs, birth preparedness and complication readiness in Burayu town, Oromia region, Ethiopia
Source: BMC Pregnancy Childbirth. 2019 Dec 21;19:515. doi: 10.1186/s12884-019-2661-4 (PMC6925843; doi:10.1186/s12884-019-2661-4)
Supplement: Supplementary file 1 — Additional file 1: Questionnaire for Men’s Knowledge on Obstetric Danger Signs, Birth Preparedness and Complication Readiness. [file 12884_2019_2661_MOESM1_ESM.dot]

# Questionnaire for Men’s Knowledge on Obstetric Danger Signs, Birth Preparedness and Complication Readiness

|  | **Background characteristics of the respondent and wife** | | | | | | |  | |
| --- | --- | --- | --- | --- | --- | --- | --- | --- | --- |
| **1** | **Questions** | | | | **Coding categories** | | | **Skip** | |
| 1.1 | Age in year at present | | | | __________________ | | |  | |
| 1.2 | Religion | | | | 1. Orthodox  2. Muslim  3. Protestant  4. Catholic  5. Other specify | | |  | |
| 1.3 | Ethnicity | | | | __________________ | | |  | |
| 1.4 | Respondent’s occupation | | | | 1. Civil servant  2. Private employee  3. Farmer  4. Merchant  5. Other | | |  | |
| 1.5 | Respondent‘s educational status | | | | 1. Illiterate  2.Read and writes  3. Primary education (1-8)  4.Secondary education (9-12)  5. Diploma and above | | |  | |
| 1.6 | Monthly house hold income in Ethiopian Birr | | | | ___________________ | | |  | |
| 1.7 | Number of wife | | | | 1.One  2.Two  3.Three  4.four and above | | |  | |
| 1.8 | Number of family size? | | | | ___________________ | | |  | |
| 1.9 | Household characteristics Residence (place of living) | | | | 1. Rural 2. Urban | | |  | |
| 1.10 | Wife’s age | | | | 1. <20 2. 20-34 3. 35-49 | | |  | |
| 1.11 | Wife/ partner have given birth in the last 12 months? | | | | 1. No 2. Yes | | | . | |
| 1.12 | Do you believe that you and your wife have good communication? | | | | 1. Yes 2. No | | |  | |
| 2 | **Attitude towards gender roles** | | | | | | |  | |
| 2.1 | Who do you believe should make decisions about health care for pregnant women? | | | | | 1. Respondent 2. Pregnant women herself 3. Respondent and wife jointly 4. Other (specify)___________ | |  | |
| 2.2 | Who do you believe should make decisions about major household purchases? | | | | | 1. Respondent 2. Pregnant women herself 3. Respondent and wife jointly 4. Other (specify)_____ | |  | |
| 2.3 | Who do you believe should make decisions about visits to health facility during pregnancy and delivery? | | | | | 1. Respondent 2. Pregnant women herself 3. Respondent and wife jointly 4. Other (specify)_______ | |  | |
| 3 | **Cultural and traditional beliefs** | | | | | | |  | |
| 3.1 | Do you believe that pregnancy is a woman affair that does not need husband involvement? | | | | | | 1. Yes 2. No 3. Don’t know |  | |
| 3.2 | Do you believe that child birth is a natural phenomenon that does not need husband involvement? | | | | | | 1. Yes 2. No 3. Don’t know |  | |
| 3.3 | Do you believe that a husband need to discuss with a doctor or health care provider in issues concerning pregnancy and delivery of his wife? | | | | | | 1. Yes 2. No 3. Don’t know |  | |
| 3.4 | What do you think about the attitude of health workers towards men who accompany their wives to health facility to seek care? | | | | | | 1. Cooperative and welcoming 2. Uncooperative and harsh 3. Do not know |  | |
| 3.5 | What do people talk about men who  escort their wives to the health unit for  ANC, delivery and postnatal care? | | | | | | 1. Good and encouraging 2. Bad and discouraging 3. Do not know |  | |
| **4** | **Health and health service related issues** | | | | | | |  | |
| 4.1 | How far is the nearest health facility offering ANC, delivery and postnatal care services from your home? | | | | | | 1. Less than 5 km 2. More than 5km |  | |
| 4.2 | How do you evaluate the quality of care for ANC, delivery and PNC services in the health unit nearest to your home? | | | | | | 1. Poor 2. Good 3. Very good 4. Excellent 5. Don’t know |  | |
| 4.3 | Do you believe that any pregnant woman is susceptible to complications during child birth? | | | | | | 1. Yes 2. No 3. Do not know |  | |
| 4.4 | Do you believe that giving birth at health facility has a better outcome than giving birth at home? | | | | | | 1. Yes 2. No 3. Do not know |  | |
| 5 | **Questions from 501-508 are about some events that happened during the recent pregnancy and child birth.** | | | | | | |  | |
| 5.1 | Where did your wife give birth to the recent baby? | | | | | | 1. Home 2. Health post 3. Health center 4. Hospital 5. Other (Specify)______ |  | |
| 5.2 | Why didn’t your wife deliver in a health facility? | | | | | | 1. Cost too much 2. Facility not open 3. Too far No transportation 4. Don’t trust facility/poor 5. Quality service 6. No female care provider 7. at facility 8. Husband did not allow 9. Relatives did not allow 10. Not necessary 11. Not customary 12. Other (specify)______ | Skip Q.5.3 if the wife delivered in health facility | |
| 5.3 | What was the outcome of birth related to your wife in the recent delivery? | | | | | | 1. Give birth without complication 2. Deliver with complication 3. Died when she gave birth |  | |
| 5.4 | Did your wife’s most recent birth result in a baby that was born alive? | | | | | | 1. Yes 2. No |  | |
| 5.5 | Did your wife give birth to a single baby, twins or more? | | | | | | 1. Single baby 2. Twins 3. Triplets or more |  | |
| 5.6 | What was the actual mode of delivery in the recent delivery? | | | | | | 1. Vaginal 2. Cesearean section |  | |
| 5.7 | While your wife was pregnant, did you know the expected date of her delivery? | | | | | | 1. Yes 2. No |  | |
| 5.8 | Who have made the plan of where to deliver from? | | | | | | 1. Respondent only 2. Respondent and wife both 3. Wife only 4. No body |  | |
| **6** | **Knowledge of Danger Signs during Pregnancy and Labour by Spouse/Male Partners** | | | | | | | | |
| 6.1 | Could you mention the Danger Signs in Pregnancy | | | 1. Vaginal bleeding 2. Severe headache 3. Blurred vision 4. General body weakness Convulsion 5. Swollen leg and face 6. High fever 7. Loss of consciousness 8. Nausea and vomiting 9. Difficulty breathing 10. Severe weakness 11. Severe abdominal pain 12. Accelerated and reduced fetal movement 13. Water breaks without labour | | | | |  |
| 6.2 | Could you mention the Danger Signs in Labour? | | | 1. Severe vaginal bleeding Severe headache 2. Frequent urination 3. Convulsion 4. High fever 5. Loss of consciousness 6. Painful uterine contraction Labour lasting greater than 12 Hours 7. Placenta not delivered after 30 minutes 8. Constipation 9. Could a woman die from this problem | | | | |  |
| 6.3 | Could you mention Newborn Care/Danger Signs of new born? | | | 1. Difficult or fast breathing Jaundice 2. Poor sucking or feeding Bleeding or discharge from the umbilicus 3. Baby very small 4. Convulsions and spasms rigidity Pinkish skin Lethargy/unconsciousness 5. Red or swollen eyes with pus | | | | |  |
| 6.4 | Where do you get about the information on Danger signs of pregnancy, labor and delivery? | | | 1. Media 2. Health providers 3. My wife 4. Others specify | | | | |  |
| **7** | **Knowledge of husbands on BP/CR** | | | | | | | |  |
| 7.1 | What kind of preparation and readiness needed for delivery (BP/CR)? | | 1. Arranging for postpartum cultural food expenses 2. Saving money for Mother’s health care 3. Identifying a mode of transportation 4. Identifying place of delivery 5. Clean clothes & other materials for Baby/Mother’s 6. Savings for emergencies Awareness an emergency & it immediately action 7. Arrangement for skilled birth assistance 8. Identifying decision maker for emergency 9. Arranging blood donors Identifying date of birth 10. Prevention of HIV mother to child | | | | | |  |
| 7.2 | Where do you get about the information on Danger signs of pregnancy, labor and delivery? | | 1. Media 2. Health providers 3. My wife 4. Others specify | | | | | |  |
| **8** | **Level of involvement** | | | | | | | |  |
| 8.1 | Which kind of preparation you attempt for the recent delivery? | 1. Arranging for postpartum cultural food expenses 2. Saving money for Mother’s health care 3. Identifying a mode of transportation 4. Identifying place of delivery 5. Clean clothes & other materials for Baby/Mother’s 6. Savings for emergencies Awareness an emergency & it immediately action 7. Arrangement for skilled birth assistance 8. Identifying decision maker for emergency 9. Arranging blood donors Identifying date of birth 10. Prevention of HIV mother to child | | | | | | |  |
| 8.2 | During antenatal care | 1. Gives permission only 2. Money for transport/drugs 3. Personally accompanies 4. Donates blood | | | | | | |  |
| 8.3 | During Delivery | 1. Gives permission only 2. Money for transport/drugs 3. Personally accompanied 4. Donates blood | | | | | | |  |
| 8.4 | Postnatal care | 1. Gives permission only 2. Money for transport/drugs 3. Personally accompanies 4. Donates blood | | | | | | |  |
